# The Causal Relationship Between Gut Microbiomes, Inflammatory Mediators, and Traumatic Brain Injury in Europeans: Evidence from Genetic Correlation and Functional Mapping Annotation Analyses

**DOI:** 10.3390/biomedicines13030753

**Published:** 2025-03-20

**Authors:** Bingyi Song, Youjia Qiu, Zilan Wang, Yuchen Tao, Menghan Wang, Aojie Duan, Minjia Xie, Ziqian Yin, Zhouqing Chen, Chao Ma, Zhong Wang

**Affiliations:** 1 Department of Neurosurgery, The First Affiliated Hospital of Soochow University, Suzhou 215006, China; jsdhsby@126.com (B.S.); qiu_youjia@163.com (Y.Q.); zlwang_1996@163.com (Z.W.); zqchen6@163.com (Z.C.); 2 Suzhou Medical College, Soochow University, Suzhou 215002, China

**Keywords:** gut microbiome, traumatic brain injury, mendelian randomization, inflammatory factor, immune cell, mapped genes

## Abstract

**Background:** The gut microbiome (GM) has been reported to play a role in traumatic brain injury (TBI). To investigate the causal relationship between GMs, inflammatory mediators, and TBI, a comprehensive Mendelian randomization (MR) analysis was conducted. **Methods:** We utilized Genome-Wide Association Study (GWAS) summary statistics to examine the causal relationships between GM and TBI. To assess the potential causal associations between GM and TBI, we employed the inverse-variance-weighted, MR-Egger, and weighted median methods. Mediation analysis was used to assess the possible mediating factors. Several sensitivity analyses methods were implemented to verify the stability of the results. Additionally, we utilized FUMA GWAS to map single-nucleotide polymorphisms to genes and conduct transcriptomic MR analysis. **Results:** We identified potential causal relationships between nine bacterial taxa and TBI. Notably, class *Methanobacteria*, family *Methanobacteriaceae*, and order *Methanobacteriales* (*p* = 0.0003) maintained a robust positive correlation with TBI. This causal association passed false discovery rate (FDR) correction (FDR < 0.05). Genetically determined 1 inflammatory protein, 30 immune cells and 3 inflammatory factors were significantly causally related to TBI. None of them mediated the relationship between GMs and TBI. The outcome of the sensitivity analysis corroborated the findings. Regarding the mapped genes of significant GMs, genes such as *CLK4*, *MTRF1*, *NAA16*, *SH3BP5*, and *ZNF354A* in class *Methanobacteria* showed a significant causal correlation with TBI. **Conclusions:** Our study reveals the potential causal effects of nine GMs, especially *Methanogens* on TBI, and there was no link between TBI and GM through inflammatory protein, immune cells, and inflammatory factors, which may offer fresh insights into TBI biomarkers and therapeutic targets through specific GMs.

## 1. Introduction

Traumatic brain injury (TBI), resulting from head trauma, constitutes a significant source of morbidity and mortality, encompassing conditions such as skull fractures, intracranial hematomas, and brain contusions [1]. Presently, global estimates indicate that more than 50 million individuals experience TBI annually, with incidence and prevalence rates on the increase over the past three decades [2]. Although TBI was once viewed as an acute ailment, it is now widely recognized as a chronic condition with the potential to affect various bodily systems [3]. Reports indicate that individuals with moderate-to-severe TBI often grapple with long-term functional impairment [4]. These injuries not only entail a loss of health and disability for affected individuals and their families, but also pose a burden on healthcare systems and economies due to diminished productivity and elevated healthcare expenses [5]. Current approaches to neurotrauma primarily emphasize prevention, trauma treatment, and post-trauma patient management. Nonetheless, there remains a lack of pertinent treatment options for its chronic progression [6]. An urgent need exists for an approach to mitigate the risk of neuropsychiatric and neurodegenerative disorders by enhancing the mechanisms addressing secondary biological damage that persists after neurotrauma.

In contemporary times, an expanding body of evidence underscores the strong association between gut microbiota (GM) and human health [7]. This correlation is particularly pronounced within the central nervous system (CNS), where GM composition significantly influences CNS development, function, and overall well-being [8]. Commensal GM actively communicate with the brain to regulate neurotransmission, neurogenesis, and neuroinflammation [9]. These intricate interactions between the GM and the brain are collectively known as the microbiota–gut–brain axis. The brain–gut axis encompasses bidirectional communication pathways, where neuroinflammation and neurodegeneration triggered by TBI can affect gut function. Conversely, subsequent gut inflammation exacerbates systemic inflammation, and worsens the neuropathological and neurobehavioral consequences of TBI [10]. Furthermore, multiple studies have provided evidence that TBI leads to GM dysbiosis. For instance, Houlden et al. report changes in the phylum *Mycobacteria*, *Porphyromonas*, and *Aspergillus* that correlate with the severity of TBI [11]. This dysbiosis is linked to chronic neuroinflammation after TBI, underscoring a robust association and regulation between the GM and the brain [12]. Investigation of the microbiota-gut–brain axis harbors potential for advancements in TBI, including its related disorders, diagnosis, and treatment. Neuroinflammation plays an integral role in the above process. Moreover, neuroinflammation is mostly mediated by immune cells such as microglia and macrophages [13]. We, therefore, speculate that inflammatory factors may play a mediating role between TBI and GM. However, further investigation is warranted to understand the modulation of GM on the immediate and enduring biological outcomes of neurotrauma.

It is important to note that a majority of studies exploring the relationship between GM and TBI involve animal experiments, and there is a dearth of relevant clinical evidence supported by randomized controlled trials. Additional research is imperative to establish whether a causal relationship exists between these two variables. The Mendelian randomization (MR) study is a statistical methodology used to assess the causal relationship between an exposure and its associated outcome. This is achieved by employing single-nucleotide polymorphisms (SNPs) with strong associations in the context of exposure factors, functioning as instrumental variables (IVs). This method enables the mitigation of confounding factors and accounts for the potential of reverse causality. MR has been employed to investigate potential causative links between GM and neurological disorders such as stroke [14] and Alzheimer’s disease [15], highlighting the connection between the gut–brain axis. Functional mapping and annotation (FUMA) provide a robust platform for examining the genetic underpinnings of complex traits, thereby unveiling the intricate links between genetic variation and phenotype [16]. Consequently, our study aimed to conduct an MR analysis, utilizing the genome-wide association study (GWAS) dataset in conjunction with FUMA analysis, to elucidate the correlation between GM and TBI, thereby offering fresh insights for TBI treatment.

## 2. Materials and Methods

### 2.1. Study Design

**Figure 1:**
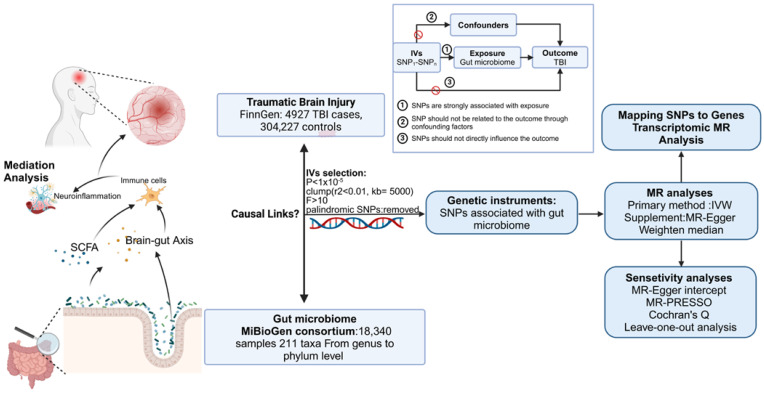
Flowchart of the present MR study and major assumptions (created with biorender.com). MR, Mendelian randomization; GWAS, genome-wide association study; SNPs, single-nucleotide polymorphisms; IVW, inverse-variance-weighted; LD, linkage disequilibrium; MR-PRESSO, MR pleiotropy residual sum and outlier.

To examine the causal relationship between GM and TBI, we employed MR techniques. The study design is illustrated in Figure 1. Three fundamental assumptions must be satisfied in MR analysis. First, SNPs selected as genetic variants should exhibit a significant association with the exposure. Second, SNPs should demonstrate no connection to the outcome through confounding factors. Last, SNPs should not directly influence the outcome. The methodology [17] of this MR analysis aligns with the STROBE-MR guidelines and has been detailed in a prior study [18].

### 2.2. Data Sources

GWAS summary-level data from the GM were derived from the extensive MiBioGen study, the most comprehensive genome-wide meta-analysis to date [19]. This study meticulously curated and analyzed data from 24 cohorts, encompassing 18,340 individuals, combining information on 16S fecal microbiome and genome-wide genotypes. The technical terms will be defined upon first use, and the text will maintain an objective tone with clear, concise sentences for a logical flow of information. Academic conventions, including precise formatting, citation, and language, will be observed. Data on 211 gut microbial taxa across five taxonomic levels (from genus to phylum) were accessed. Adjustment was made for sex, age, study-specific covariates, and the primary genetic principal components reflecting population stratification [19]. GWAS statistics for TBI were obtained from the dataset published by FinnGen Research in January 2017, serving as the source for outcome events in 309,154 individuals of European descent, and encompassed 8304 cases with TBI (diagnosed according to the International Classification of Diseases 10) and 445,429 controls. Genetic data for cytokines were from a previous GWAS (8337 individuals) and included 41 cytokines.

### 2.3. Identification of IVs

Given the limited number of cases meeting the stringent threshold of *p* < 5 × 10^−8^ in the initial IV identification, IVs with a threshold of *p* < 1 × 10^−5^ were selected to bolster the available IVs for GM and immune cells. For inflammatory proteins and inflammatory factors, we chose *p* < 5 × 10^−6^ as the threshold. Additionally, clumping function parameters were set to r^2^ < 0.001 and distance > 10,000 kb to ensure the independence of each IV and mitigate the influence of linkage disequilibrium (LD) on the random allocation of alleles. Subsequently, SNPs with palindromic sequences and those absent from the results were removed. Finally, F-values (calculated as beta^2^/se^2^) were employed to discard the effects of weak IVs, eliminating those with F statistics < 10. IVs with an F statistic below 10 were deemed weak and excluded.

### 2.4. Statistical Analyses

To assess the causal association between GM and TBI, we primarily utilized the inverse-variance-weighted (IVW) method, supplemented by the weighted median, weight mode, simple mode, and MR-Egger regression methods [20,21]. IVW assumes that all SNPs representing genetic variation serve as valid IVs and collectively exhibit zero overall bias. Cochran’s Q test and I^2^ statistics were applied to evaluate heterogeneity. MR-Egger allows for the existence of horizontal pleiotropy in more than 50% of the IVs [22]. However, incorporating an intercept term in MR-Egger regression may yield estimates of causality that are statistically less efficient [22]. In contrast, the weighted median method produces more accurate estimations when more than 50% of IVs are invalid [20]. Odds ratios (OR) and their corresponding 95% confidence intervals (CIs) were used to present the MR results, with significance set at *p* < 0.05. Additionally, the *p*-values were adjusted using the false discovery rate (*p*-FDR) to account for multiple comparisons [23]. A causal association was confirmed when *p*-FDR < 0.05 in the IVW method, and a suggested association was considered when *p* < 0.05 but *p*-FDR > 0.05 in the IVW method.

### 2.5. Mediation Analysis

Following the assessment of causal effects through the utilization of a two-sample analysis, the selected GM, inflammatory protein, immune cells, and inflammatory factors with a significant causal impact on TBI were incorporated into the mediation analysis. In the event that a causal effect was identified between GM, inflammatory protein, immune cells, and inflammatory factors, an investigation was conducted to ascertain whether cytokines act as mediators in the pathway from GM to TBI.

### 2.6. Mapping SNPs to Genes and Transcriptomic MR Analysis

To explore the relationship between GM and TBI, we identified significant SNPs from each GM and included them as primary SNPs in the FUMA GWAS [24]. These SNPs were annotated to genes using FUMA’s SNPGENE tool. To further examine the role of genes associated with positive GMs in TBI, we conducted a transcriptomic MR analysis. Cis-eQTL data for these genes were obtained from the eQTLGen Consortium (https://eqtlgen.org/), covering 16,987 genes derived from 31,684 blood samples, predominantly from a European cohort of healthy individuals [25]. To avoid excluding potential causal variants due to overly stringent criteria, we applied an LD-based clumping process with an r^2^ threshold of <0.1 for the eQTLs. The resulting SNPs were used as IVs in the MR analysis to assess their association with TBI. Statistical significance was determined using FDR correction, with a threshold of FDR < 0.05.

### 2.7. Ethical Approval

The genetic data utilized in this study are publicly accessible and de-identified. These data previously obtained approval from an ethics committee, thus negating the need for further ethical clearance in this study.

## 3. Results

### 3.1. Details of GMs

In total, 211 GMs were included in this research, spanning biological levels from phylum to genus. Fifteen bacterial traits with unknown characteristics were excluded, leaving a total of 192 bacterial traits from the FinnGen datasets for inclusion in the MR analysis. Ultimately, nine GMs were identified to exhibit a causal link with TBI. The F statistics for the selected IVs exceeded 10, effectively eliminating bias induced by weak IVs. Detailed information about the IVs can be found in Supplementary Tables S1–S4.

### 3.2. MR Estimates

**Figure 2:**
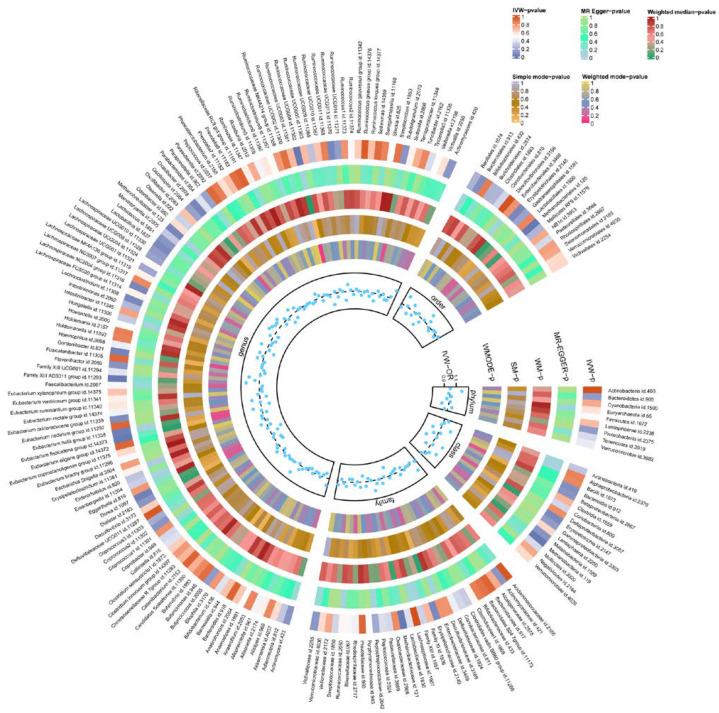
Heatmap of the MR estimates of GM and TBI. The different colors represent the *p*-values derived from the analysis of GM and TBI causality using each method. GM, gut microbiome; TBI, traumatic brain injury; IVW, inverse-variance-weighted method; WM, weighted median; OR, odds ratios; SM, simple mode; WMODE, weighted mode.

Through preliminary analysis, we found that nine GMs were significantly associated with TBI (Table 1). For instance, class *Methanobacteria* (OR = 1.175, 95% CI = 1.077 to 1.282, *p* = 0.0003), order *Methanobacteriales* (OR = 1.175, 95% CI = 1.077 to 1.282, *p* = 0.0003), family *Methanobacteriaceae* (OR = 1.175, 95% CI = 1.077 to 1.282, *p* = 0.0003), genus *Eubacterium fissicatena group* (OR = 1.143, 95% CI = 1.038 to 1.258, *p* = 0.006), and genus *Family XIII AD3011* group (OR = 1.222, 95% CI = 1.057 to 1.412, *p* = 0.007) were associated with an increased risk of TBI. Conversely, genus *Dorea* (OR = 0.791, 95% CI = 0.637 to 0.983, *p* = 0.034), genus *Eubacterium hallii group* (OR = 0.858, 95% CI = 0.751 to 0.980, *p* = 0.024), genus *Gordonibacter* (OR = 0.920, 95% CI = 0.849 to 0.998, *p* = 0.046), and genus *Ruminococcaceae UCG004* (OR = 0.816, 95% CI = 0.718 to 0.928, *p* = 0.002) were associated with a reduced risk of TBI. Of 14 GMs, class *Methanobacteria*, family *Methanobacteriaceae*, and order *Methanobacteriales* passed FDR correction (FDR < 0.05). It is worth mentioning that although genus *Ruminococcaceae UCG004* did not pass the FDR correction, it also achieved a high significance (FDR < 0.1). Full MR estimates are presented in Figure 2 and Supplementary Table S5.

### 3.3. Sensitivity Analysis and Reverse MR Analysis

Sensitivity analyses, including MR-Egger intercept and MR-PRESSO tests, did not uncover any horizontal pleiotropy (Supplementary Table S6). Leave-one-out analysis did not detect any anomalous SNPs. The scatter plot in Supplementary Figure S1 illustrates the SNP effect on GMs, and Supplementary Figure S2 shows the results of leave-one-out analysis. The result of the funnel plot is shown in Supplementary Figure S3. In conclusion, these investigations collectively underscore the robustness and stability of the causal link between GM and TBI. We further explored reverse causality using TBI as exposure and significant GMs as outcomes. The genus *Dorea* and genus *Eubacterium hallii groups* were excluded from the analysis because the obtained SNPs < 3. As shown in Supplementary Table S7, no reverse causality was observed between TBI and GMs (*p* > 0.05), and sensitivity analysis also did not indicate any evidence of horizontal pleiotropy.

### 3.4. Mediation Analysis

**Figure 3:**
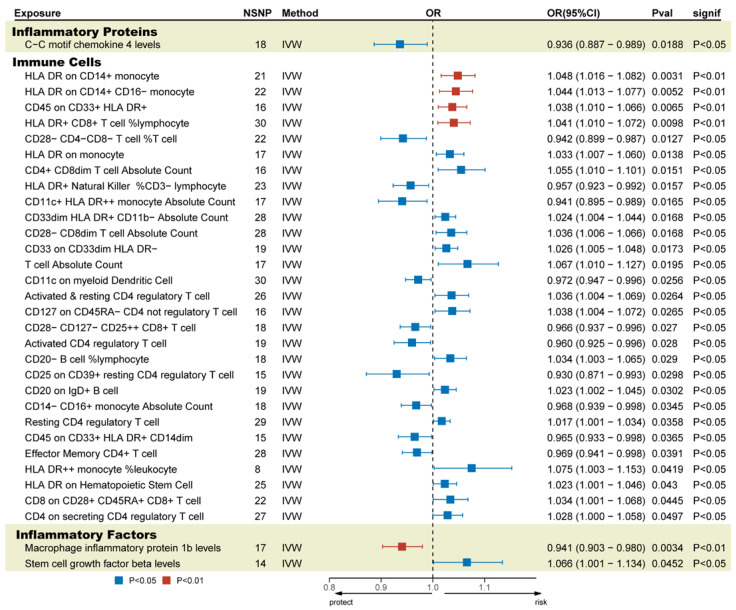
Possible mediating factors and positive results of the MR analysis of TBI. IVW, inverse-variance-weighted method; OR, odds ratios; Pval, *p*-value.

**Figure 4:**
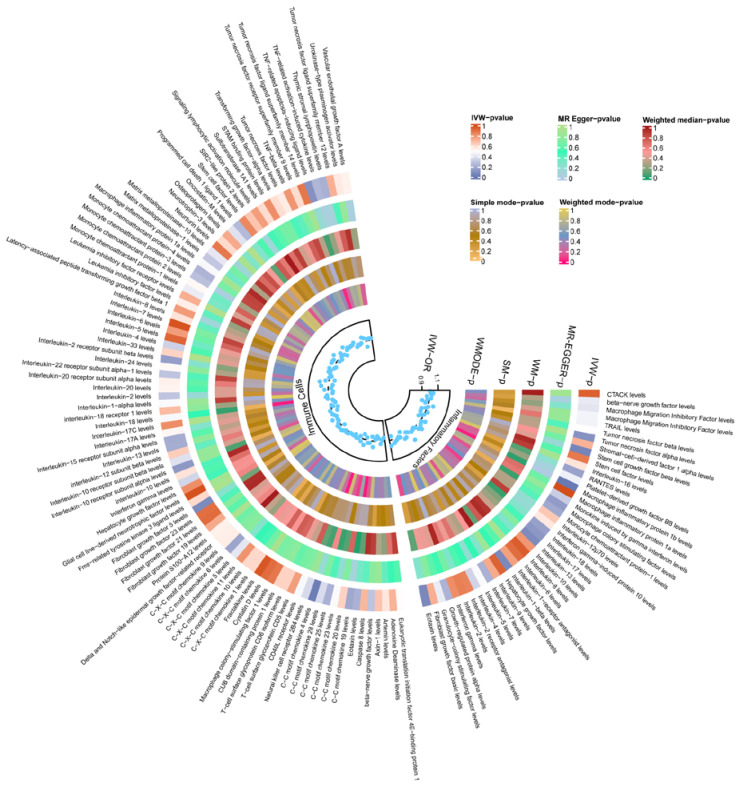
Heatmap of the MR estimates of immune cells, inflammatory factors, and TBI. TBI, traumatic brain injury; IVW, inverse-variance-weighted method; WM, weighted median; OR, odds ratios; SM, simple mode; WMODE, weighted mode.

In this study, both GM and cytokines were causally associated with TBI. Genetically determined 1 inflammatory protein, 30 immune cells, and 3 inflammatory factors were significantly causally related to TBI (Figure 3). Full MR estimates are presented in Figure 4 and Supplementary Tables S8–S10. No obvious heterogeneity or horizontal pleiotropy was found through sensitivity analyses (T). We selected the FDR-corrected positive GMs (class *Methanobacteria*, family *Methanobacteriaceae*, and order *Methanobacteriales*) for mediation analysis. Unfortunately, no mediating factors were found to play a mediating role between them and TBI (Supplementary Table S11). In order to explore more possible mediating factors, we also included genus *Ruminococcaceae UCG004* in the mediation analysis. Further analysis showed that *CD4+ CD8dim T cell Absolute Count* (OR = 0.798, 95% CI = 0.644 to 0.989, *p* = 0.039) and *CD33dim HLA DR+ CD11b- Absolute Count* (OR = 1.324, 95% CI = 1.011 to 1.734, *p* = 0.041) were significantly correlated with GM (Supplementary Table S11). However, the above factors did not pass the Sobel test and cannot be included as mediating factors.

### 3.5. Mapping SNPs to Genes and Transcriptomic MR Analysis

**Figure 5:**
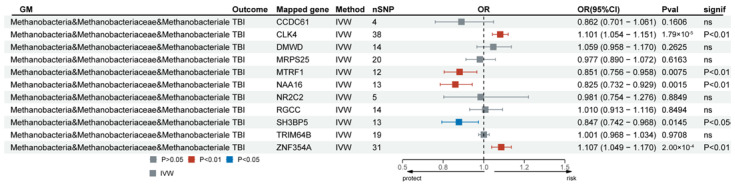
Forest plot of the mapped genes positively associated with TBI in class *Methanobacteria*. TBI, traumatic brain injury; IVW, inverse-variance-weighted method; OR, odds ratios; CI, confidence interval; ns, no significance.

IVs serving as genetic variants were functionally annotated using the FUMA GWAS tool to explore more biologically significant findings. Following verification, class *Methanobacteria*, family *Methanobacteriaceae*, and order *Methanobacteriales* shared the same mapped genes (Supplementary Table S12). Of these mapped genes, SNPs associated with 11 genes were obtained from the eQTLGen consortium (https://eqtlgen.org/). Transcriptomic MR analysis was performed on the mapped genes of class *Methanobacteria* and TBI. Among these, *CLK4*, *MTRF1*, *NAA16*, *SH3BP5*, and *ZNF354A* in class *Methanobacteria* were positively associated with TBI after FDR correction (Figure 5 and Supplementary Table S13). The heterogeneity test indicated no significant heterogeneity in the MR analysis (Supplementary Table S14). The MR-Egger intercept test provided no clear evidence of horizontal pleiotropy between the causality of *CLK4*, *MTRF1*, *NAA16*, *SH3BP5*, and *ZNF354A* in class *Methanobacteria* and TBI.

## 4. Discussion

In this study, we conducted MR analyses to probe the potential causal relationship between GMs and TBI. Our findings revealed that nine GMs exhibited associations with TBI based on extensive GWAS statistics. Notably, the class *Methanobacteria*, family *Methanobacteriaceae*, and order *Methanobacteriales* in TBI exhibited a significant causal link after FDR correction. Genus *Ruminococcaceae* also achieved a high significance. Additionally, *CLK4*, *MTRF1*, *NAA16*, *SH3BP5*, and *ZNF354A* in class *Methanobacteria* were positively associated with TBI after FDR correction. These results may provide crucial insights into the pathogenesis and therapeutic implications of GM in TBI.

A systematic review assessed the association between GM and TBI, and the studies demonstrated inconsistent changes in GM after TBI [26]. Nicholson et al. corroborated a general trend suggesting a decline in beneficial microbial species over time and an elevation in pathogenic bacteria in the presence of head injuries. This can lead to heightened intestinal permeability and an increased risk of other adverse conditions [27]. Numerous GMs have the potential to serve as TBI biomarkers, including *Clostridium*, *Prevotella*, *Ruminococcaceae*, and *Lactobacillaceae*, the abundance of which undergoes significant shifts following TBI [28]. Even in the chronic phase of TBI, patients experience shifts in their GM composition, with reductions or absence of *Prevotella* and *Mycobacterium* in the fecal microbiome of chronic TBI patients [29]. Given prior research indicating GM changes in TBI patients, fecal microbiota transplantation presents a potential treatment avenue for an improving prognosis, mitigating secondary brain damage, and enhancing functional outcomes in patients with chronic TBI [30].

Drawing from our findings and previous research, we speculate on the mechanism of GM involvement in TBI, considering the following aspects. TBI encompasses a diverse injury process leading to the generation of free radicals, metabolic disruption, neuronal excitotoxicity, and significant neuroinflammation. These processes can give rise to various behavioral, neurocognitive, and motor impairments [31,32]. However, the underlying mechanisms of the chronic processes that occur after the acute phase of TBI necessitate further exploration. Existing research reveals several potential mechanisms. For example, loss of the GM has a significant impact on microglia and regulates the transition between different microglia subpopulations. This gut–brain axis communication potentially regulates microglia-mediated central immune and neuroinflammatory dysregulation [33]. In addition, pathological processes such as excessive activation of microglia by lipopolysaccharide, impairment of intestinal barrier function, and regulation of inflammation by short-chain fatty acids (SCFAs) are closely related to GM [34]. The GM is intimately involved in the above pathologic processes.

*Ruminococcaceae*, a microbiome belonging to the phylum *Firmicutes*, was associated with a decreased risk of TBI in our MR analysis, exhibiting greater abundance in TBI [29]. The significantly altered abundance of *Ruminococcaceae* following TBI suggests its potential as a TBI biomarker, which significantly reduced after TBI [35]. From the perspective of GM metabolites, SCFA signaling, as described earlier, has been linked to blood–brain barrier (BBB) permeability, microglia polarization and function, and neurogenesis [36]. Critically ill patients and corresponding murine models consistently demonstrated a reduction in commensal bacteria that produce SCFAs [37]. Contrastingly, Opeyemi et al. showed that SCFA-producing commensal bacteria in the *Ruminococcaceae* are progressively depleted after TBI [38]. This suggests that the levels of SCFA detected in fecal samples decline following TBI. SCFA can activate sympathetic nervous system activity and reduce intestinal inflammation, and have potential direct effects on the morphology and function of microglia and macrophages by penetrating the BBB and infiltrating the CNS [39,40]. This is consistent with our results. These avenues could be valuable for mechanistic and prognostic investigation into chronic TBI.

The FDR test confirmed the causality of class *Methanobacteria*, family *Methanobacteriaceae*, and order *Methanobacteriales*, all part of the Methanogenic Archaea group found in the gut. Methanogenic Archaea group use hydrogen in the intestine to produce methane, which competes with other pathways for disposing of hydrogen in the intestine [41]. In addition, the abundance of methanogenic bacteria is negatively correlated with SCFA, and an increase in its abundance may be detrimental to the regulation of inflammation by SCFA [42]. Contrary to the above studies, inhaling methane gas or using methane saline can improve ischemia-reperfusion injury in experimental animals, or play anti-inflammatory and neuroprotective effects [43,44]. *Methanogenic Archaea group* may affect CNS inflammation through similar pathways. In short, the existing research can only indirectly reflect the relationship between methanogenic bacteria and TBI, and the direct relationship needs to be confirmed by further research.

Beyond the mechanisms described above, FUMA analysis offers new genetic-level perspectives. FUMA analysis identified five positive gene loci associated with GMs, primarily *Methanogens*, and TBI, suggesting a potential association between the two. Of these five common genes, *CLK4*, *MTRF1*, and *SH3BP5* were found to be expressed in the brain. *MTRF1* recognizes mitochondrial non-canonical termination codons (e.g., AGA and AGG) and plays a role in cognitive function and neuronal plasticity. Loss of its activity can lead to mitochondrial dysfunction [45,46]. The mitochondrial scaffold protein *SH3BP5* is involved in signaling pathways related to mitochondrial dysfunction and apoptosis [47], making it an important signaling platform for neurodegenerative diseases. Brain-specific *SH3BP5*-mediated signaling pathways are implicated in neuronal activity by influencing mitochondrial physiology through interacting kinases [48], potentially correlating with neuronal damage and susceptibility to neurological disease during TBI. While the impact of *CLK4*, *MTRF1*, *NAA16*, *SH3BP5*, and *ZNF354A* risk genes on the underlying mechanism between GMs and TBI remains uncertain, this study’s results provide valuable insights for future mechanistic investigations, as these genes may be implicated in the pathophysiological basis of GM-induced TBI.

This study possesses several strengths. First, it comprehensively examines the impact of GMs on TBI, employing an analysis method less prone to confounding and reverse causality compared with observational studies. Second, the GWAS database provides a large number of samples and strong estimated effects for each genetic variant (all F-statistics > 10), ensuring the study’s strong statistical power. Third, thorough analyses, including three MR methods and heterogeneity tests, were performed to mitigate potential pleiotropic influences and reduce type I error through FDR correction. Last, this is the first study to utilize MR analysis in combination with FUMA analysis to explore the causal relationship between GMs and TBI. Based on this article, we can further explore the tandem relationship between specific GMs and microglial subtype transformations, and the resulting neuroinflammation. While exploring the transplantation of specific GMs, we will target the GM and the biological peptides it secretes to improve the prognosis of TBI. However, the study has several limitations. The GWAS data on GMs cover a wide range of diseases and ages without population, gender, and age stratification. Additionally, the available GWAS data do not distinguish between acute and chronic phases of TBI, preventing stratification for analysis. Recent investigations into GMs have primarily focused on bacterial components, but it is worth noting that various other types of GM may harbor potential functional roles.

## 5. Conclusions

In conclusion, our MR findings support a causal relationship between GMs and TBI. The microorganisms of class *Methanobacteria*, family *Methanobacteriaceae*, and order *Methanobacteriales* may have an impact on TBI. This investigation did not yield evidence of a substantial mediating role of inflammatory proteins, immune cells, and inflammatory factors between TBI and GM. Further exploration is needed to understand the role of GMs in the pathogenesis and prevention of TBI.

## Figures and Tables

**Table 1:** The positive result of the MR estimates.

Exposure	Outcome	Method	No. SNP	*p*-Value	SE	OR (95%CI)	Cochran’s Q Test	
							Q	Q *p*-Value
class *Methanobacteria*	TBI	IVW	10	<0.001	0.045	1.175 (1.077, 1.282)	5.114	0.745
family *Methanobacteriaceae*	TBI	IVW	10	<0.001	0.045	1.175 (1.077, 1.282)	5.114	0.745
genus *Dorea*	TBI	IVW	10	0.034	0.111	0.791 (0.637, 0.983)	11.858	0.158
genus *Eubacterium fissicatena group*	TBI	IVW	9	0.006	0.049	1.143 (1.038, 1.258)	3.585	0.826
genus *Eubacterium hallii group*	TBI	IVW	14	0.024	0.068	0.858 (0.751, 0.980)	11.152	0.516
genus *Family XIII AD3011 group*	TBI	IVW	13	0.007	0.074	1.222 (1.057, 1.412)	11.241	0.423
genus *Gordonibacter*	TBI	IVW	11	0.046	0.041	0.920 (0.849, 0.998)	8.750	0.461
genus *Ruminococcaceae UCG004*	TBI	IVW	11	0.002	0.066	0.816 (0.718, 0.928)	4.931	0.840
order *Methanobacteriales*	TBI	IVW	10	<0.001	0.045	1.175 (1.077, 1.282)	5.114	0.745

TBI, traumatic brain injury; IVW, inverse-variance-weighted; SNP, single-nucleotide polymorphism; SE, standard error; OR, odds ratios; CI, confidence interval.

## Data Availability

Data is contained within the article or Supplementary Material.

## Supplementary Materials

The following supporting information can be downloaded at: https://www.mdpi.com/article/10.3390/biomedicines13030753/s1, Supplementary Figure S1: Scatter plot of the MR results; Supplementary Figure S2: Leave-one-out analysis of the MR results; Supplementary Figure S3. Funnel plot of the MR results; Supplementary Table S1: Included instrumental variables for significant taxa between TBI and GMs; Supplementary Table S2： Included instrumental variables for significant taxa between TBI and inflammatory proteins; Supplementary Table S3: Included instrumental variables for significant taxa between TBI and immune cells; Supplementary Table S4: Included instrumental variables for significant taxa between TBI and inflammatory factors; Supplementary Table S5: MR results of 192 GMs in TBI; Supplementary Table S6: Horizontal pleiotropy analysis for IVs of the nine GM taxa associated with TBI; Supplementary Table S7: Results of the reverse MR analysis; Supplementary Table S8: MR results of inflammatory proteins in TBI; Supplementary Table S9: MR results of immune cells in TBI; Supplementary Table S10: MR results of inflammatory factors in TBI; Supplementary Table S11: Results of MR analysis of GM and the possible mediating factors; Supplementary Table S12: The result of the mapped genes; Supplementary Table S13: MR analysis results of all five methods for significant genes; Supplementary Table S14: Sensitivity analysis results for significant genes.

## Abbreviations

TBI	Traumatic brain injury
MR	Mendelian randomization
GM	Gut microbiome
GWAS	Genome-Wide Association Study
IVW	Inverse-variance-weighted
WM	Weighted median
OR	Odds ratios
CI	Confidence interval
LD	Linkage disequilibrium
