# Supplementary material for: The Causal Relationship Between Gut Microbiomes, Inflammatory Mediators, and Traumatic Brain Injury in Europeans: Evidence from Genetic Correlation and Functional Mapping Annotation Analyses
Source: Biomedicines. 2025 Mar 20;13(3):753. doi: 10.3390/biomedicines13030753 (PMC11939942; doi:10.3390/biomedicines13030753)
Supplement: Supplementary file 1 [file biomedicines-13-00753-s001.zip › Supplementary Figures.pdf]

Supplementary Material

The causal relationship between gut microbiomes, inflammatory mediators, and traumatic brain injury in Europeans: evidence from genetic correlation and Functional

Mapping Annotation Analyses

Bingyi Song[1,†], Youjia Qiu[1,†], Zilan Wang[1], Yuchen Tao[2], Menghan Wang[1], Aojie Duan[1], Minjia Xie[1], Ziqian Yin[1], Zhouqing Chen[1], Chao Ma[1,*], Zhong Wang[1,*]

[1]Department of Neurosurgery, The First Affiliated Hospital of Soochow University, Suzhou 215006, China

[2]Suzhou Medical College, Soochow University, Suzhou 215002, China

*** Correspondence:** machaoss@163.com (C.M.); wangzhong761@163.com (Z.W.)

† These authors contributed equally to this work.

Figure Legends

Supplementary Figure S1. Scatter plot of MR results. (A) class Methanobacteria; (B) family Methanobacteriaceae; (C) genus Dorea; (D) genus Eubacterium fissicatena group; (E) genus Eubacterium hallii group; (F) genus Family XIII AD3011 group; (G) genus Gordonibacter; (H) genus Ruminococcaceae UCG004; (I) order Methanobacteriales

Supplementary Figure S2. Leave-one-out analysis of MR results. (A) class Methanobacteria; (B) family Methanobacteriaceae; (C) genus Dorea; (D) genus Eubacterium fissicatena group; (E) genus Eubacterium hallii group; (F) genus Family XIII AD3011 group; (G) genus Gordonibacter; (H) genus Ruminococcaceae UCG004; (I) order Methanobacteriales

Supplementary Figure S3. Funnel plot of MR results. (A) class Methanobacteria; (B) family Methanobacteriaceae; (C) genus Dorea; (D) genus Eubacterium fissicatena group; (E) genus Eubacterium hallii group; (F) genus Family XIII AD3011 group; (G) genus Gordonibacter; (H) genus Ruminococcaceae UCG004; (I) order Methanobacteriales

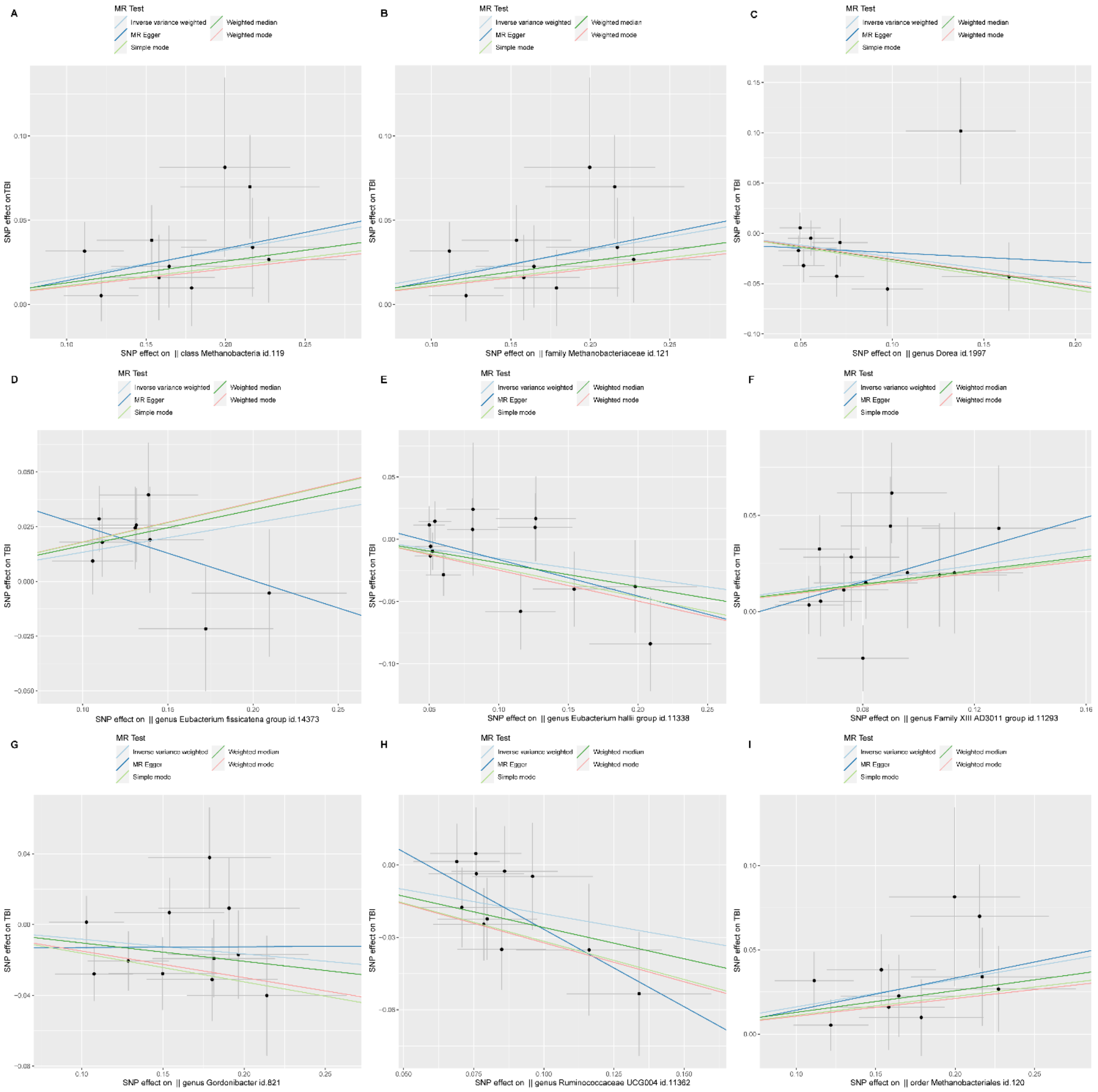

Supplementary Figure S1. Scatter plot of MR results. (A) class Methanobacteria; (B) family Methanobacteriaceae; (C) genus Dorea; (D) genus Eubacterium fissicatena group; (E) genus Eubacterium hallii group; (F) genus Family XIII AD3011 group; (G) genus Gordonibacter; (H) genus Ruminococcaceae UCG004; (I) order Methanobacteriales

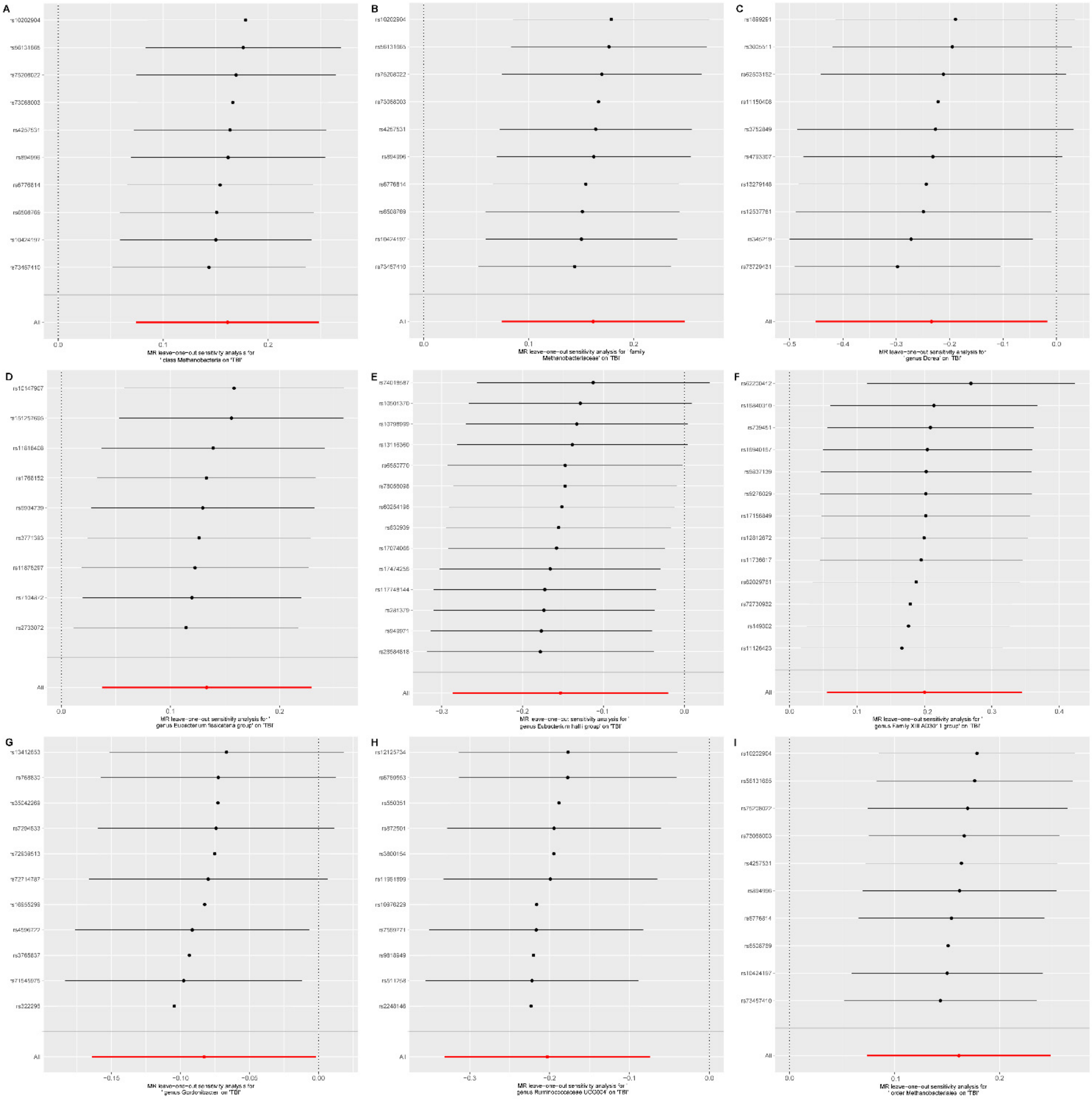

Supplementary Figure S2. Leave-one-out analysis of MR results. (A) class Methanobacteria; (B) family Methanobacteriaceae; (C) genus Dorea; (D) genus Eubacterium fissicatena group; (E) genus Eubacterium hallii group; (F) genus Family XIII AD3011 group; (G) genus Gordonibacter; (H) genus Ruminococcaceae UCG004; (I) order Methanobacteriales

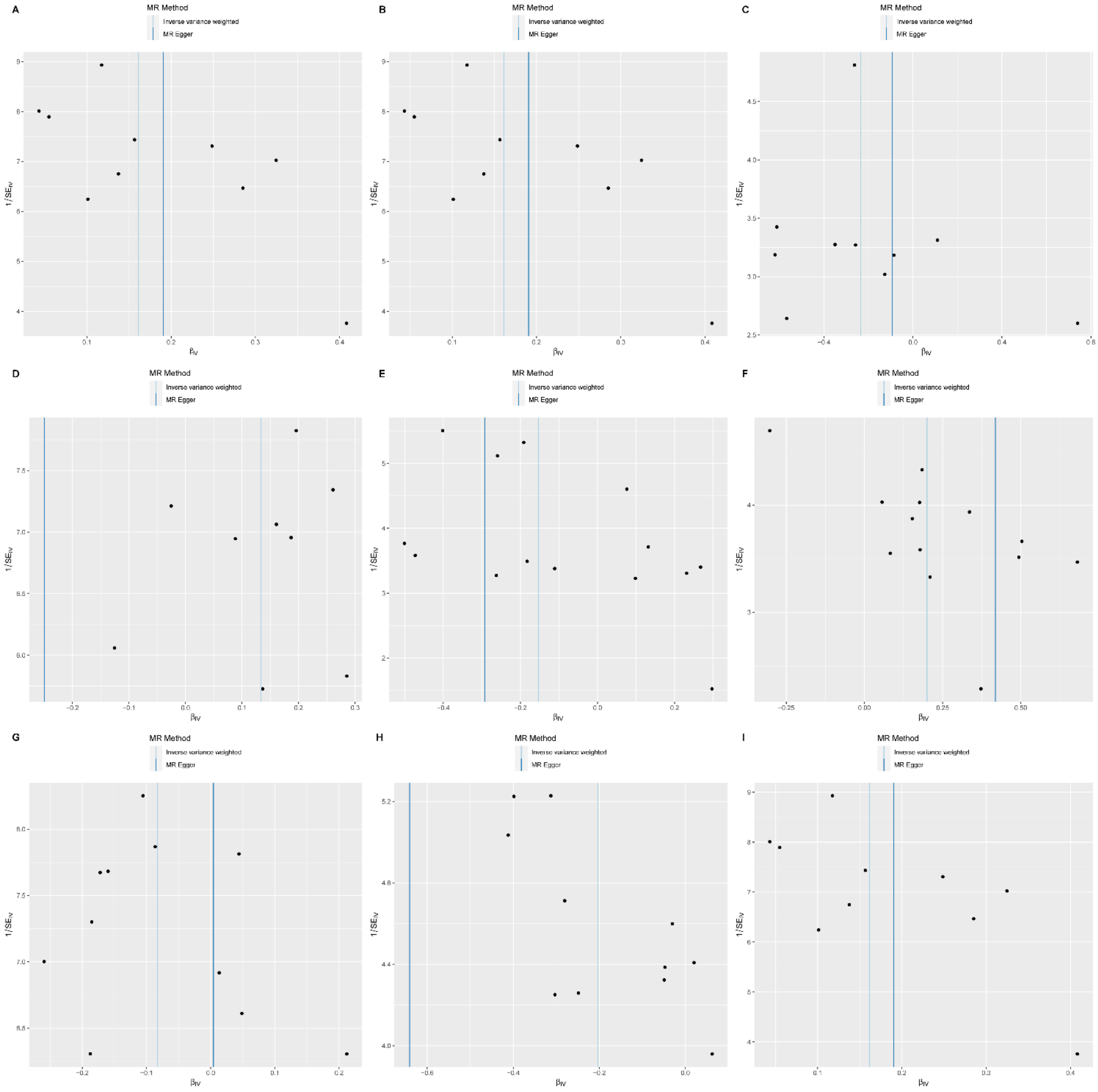

Supplementary Figure S3. Funnel plot of MR results. (A) class Methanobacteria; (B) family Methanobacteriaceae; (C) genus Dorea; (D) genus Eubacterium fissicatena group; (E) genus Eubacterium hallii group; (F) genus Family XIII AD3011 group; (G) genus Gordonibacter; (H) genus Ruminococcaceae UCG004; (I) order Methanobacteriales
